# Improving the estimation accuracy of rice leaf protein nitrogen using data augmentation, explainable machine learning, and UAV hyperspectral imagery

**DOI:** 10.3389/fpls.2026.1760799

**Published:** 2026-04-24

**Authors:** Yiping Peng, Yuting Tu, Yanggui Xu, Wenliang Zhong, Zhuxian Li, Jianyi Liang, Jichuan Huang

**Affiliations:** 1 Institute of Agricultural Resources and Environment, Guangdong Academy of Agricultural Sciences, Guangzhou, China; 2 Key Laboratory of Plant Nutrition and Fertilizer in South Region, Ministry of Agriculture and Rural Affairs of the People’s Republic of China, Guangzhou, China; 3 Guangdong Key Laboratory of Nutrient Cycling and Farmland Conservation, Guangzhou, China

**Keywords:** data augmentation, interpretability, nitrogen monitoring, precision agriculture, UAV hyperspectral imagery

## Abstract

Efficiently estimating the protein nitrogen content of rice leaves (LPN) is crucial for monitoring the nutritional health of rice and guiding precision fertilization based on requirements. Unmanned aerial vehicle (UAV)-acquired hyperspectral imagery is a key tool for estimating rice nitrogen content. Previous studies have demonstrated the potential of machine learning models for this task. However, these models typically require substantial data for supervised training to ensure high performance and generalizability. Acquiring a large sample size is challenging due to weather conditions, high collection costs, and other factors. Moreover, machine learning models have low interpretability. Enhancing it is vital for understanding the model’s decision-making. To address these issues, we utilized the Wasserstein-generative adversarial network (WGAN) algorithm to expand the sample dataset. This method employs statistical regression (multiple linear regression (MLR) and partial least squares regression (PLSR)) and machine learning (support vector machines (SVM) and K-nearest neighbor (KNN)) algorithms to establish an estimation model for the LPN. The Shapley Additive exPlanations (SHAP) method was used to analyze the contributions of the input features to LPN estimation. An experiment was conducted at the National Agricultural Science and Technology Park, Guangzhou, Baiyun District, Guangdong, China. The model based on the KNN provided the optimum estimation performance, and the model accuracy was improved by adding the augmented dataset, resulting in a 10.39% improvement in the R^2^ value. The SHAP values revealed that B_775.6_, double-peak canopy nitrogen index (DCNI), and MERIS terrestrial chlorophyll index (MTCI) were the core variables for LPN estimation. These findings provide significant references for precision fertilization and improving nitrogen use efficiency in rice cultivation.

## 1 Introduction

Nitrogen metabolism is closely related to the growth and development of rice. The science-based application of nitrogen fertilizer is required to achieve high rice yield and quality (Wang et al., 2023). Nitrogen exists in plant tissues predominantly as protein and non-protein nitrogen. The dynamic changes in the content and proportion of these two forms are the core characteristics of the plant’s nitrogen metabolism (Shi et al., 2015). Research has shown that the proportion of non-protein nitrogen is relatively high during the rapid growth stage of plants. Protein nitrogen becomes dominant in the reproductive growth stage, especially after reaching maturity. Nitrogen deficiency can reduce protein synthesis in plants, accelerate protein decomposition in old organs, and significantly decrease the protein nitrogen content. Excessive nitrogen increases the proportion of non-protein nitrogen. The content changes of different nitrogen components depend on the crop type, cultivation conditions, and nitrogen nutritional status. The nitrogen component in an organ at different developmental stages is closely related to the plant’s physiological state (Yang et al., 2009). Therefore, obtaining information on plant protein nitrogen content is crucial for understanding plant nitrogen absorption, transport, and metabolism, as well as for diagnosing the crop’s physiological status and nitrogen nutritional status (Li et al., 2024).

Acquiring hyperspectral imagery from unmanned aerial vehicles (UAVs) has the advantages of high spectral resolution and high detection sensitivity. This method has become an important approach for monitoring the nitrogen status of rice (Li et al., 2024; Wang et al., 2024). Most studies have focused on total nitrogen estimation, whereas the spectral monitoring of different nitrogen components, especially leaf protein nitrogen, remains in the exploratory stage (Yang et al., 2009; Li et al., 2024). Inversion studies of rice nitrogen using UAV hyperspectral technology have utilized statistical regression and machine learning models (Li et al., 2024). The rapid development of machine learning models has increased their use in vegetation attribute mapping. Studies have shown that they are superior to statistical regression methods in nitrogen hyperspectral inversion (Wang et al., 2024). However, machine learning models usually require a large amount of data for effective training to ensure high model performance and generalization ability. Their applicability is limited when data are scarce (Yang et al., 2022; Wang et al., 2025). Although machine learning models, such as support vector machines and K-nearest neighbor, generally require less data than deep learning models, their performance in hyperspectral applications remains limited when the sample size is small relative to the dimensionality of the feature space. In this study, we define a “small sample size” as a dataset containing fewer than 200 samples. UAV hyperspectral data acquisition is expensive and weather-dependent, making it challenging to obtain a large sample size. These drawbacks have limited the use of machine learning methods for the rapid evaluation of rice nitrogen.

Machine learning models are data-driven, have strong nonlinear fitting capabilities, and can automatically determine the nitrogen content of crops and changes over time. Although machine learning models can provide higher estimation accuracy than statistical regression methods, they are black box models, limiting their interpretability and credibility (Ma et al., 2025; Yang et al., 2022). It is challenging to determine potential interactions and causal relationships between variables and clarify the model’s logic. Therefore, improving the interpretability of machine learning models and quantifying the features’ contributions to model estimations are critical research directions.

This paper proposes a method for estimating the protein nitrogen content of rice leaves (LPN) using data augmentation and model interpretability. Extreme gradient boosting (XGBoost) and correlation analysis are used to select the variables. The Wasserstein-generative adversarial network (WGAN) algorithm is utilized to expand the sample set to alleviate data scarcity. Partial least squares regression (PLSR), multiple linear regression (MLR), support vector machine (SVM), and K-nearest neighbor (KNN) are used to construct relationship models between feature variables and the LPN. The model accuracies are compared to determine the optimal estimation model. The Shapley Additive exPlanations (SHAP) algorithm is used to analyze the features’ contributions to the model’s output. This strategy improves model interpretability and provides insights for optimizing the model.

## 2 Materials and methods

**Figure 1:**
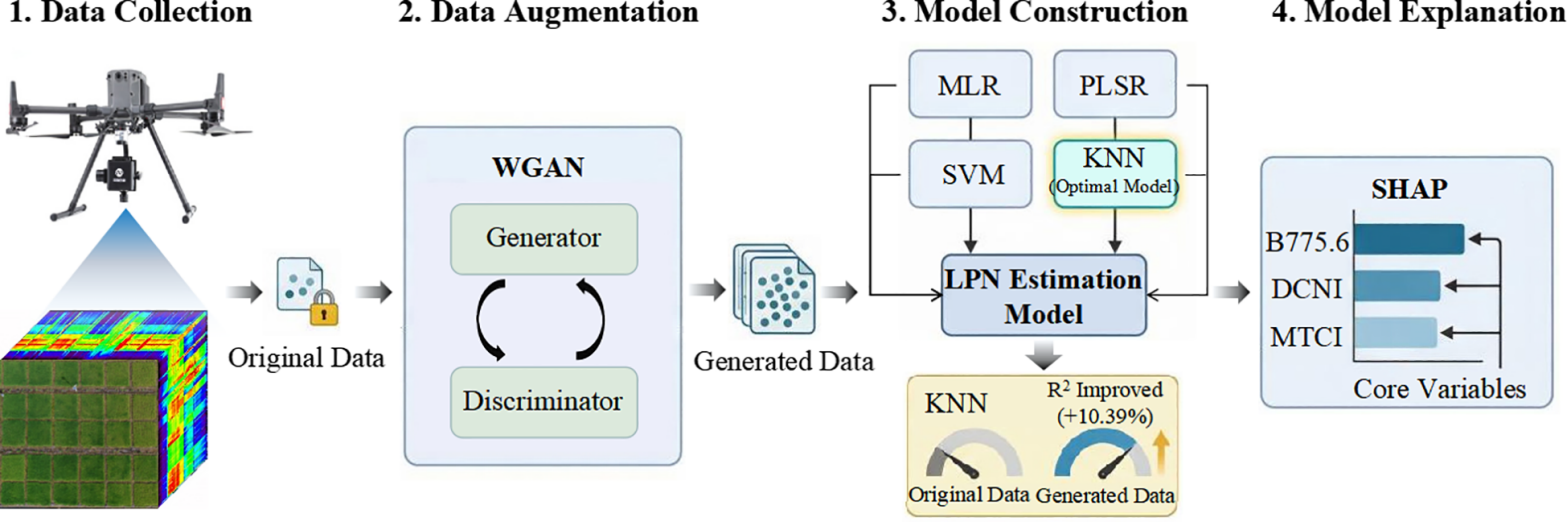
The flow chart of LPN estimation.

In this study, a method for estimating rice leaf protein nitrogen (LPN) using UAV hyperspectral imagery was developed. **Figure 1** shows the flowchart of the proposed method. The methodological framework consists of data collection, data augmentation using WGAN, construction of LPN estimation models, and model interpretation using SHAP.

### 2.1 Field experimental design

**Figure 2:**
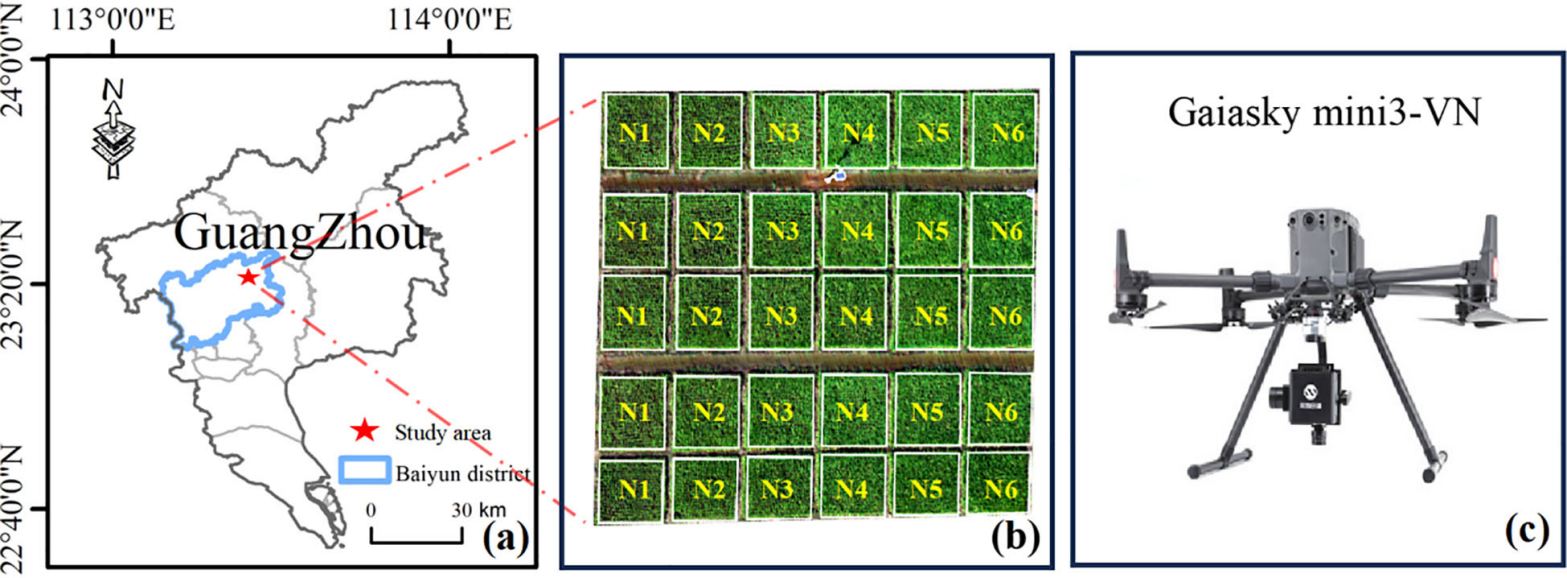
The geographical location of the experimental station and the instruments. **(a)** The location of the study area, **(b)** the layout of the experimental blocks, and **(c)** the Gaiasky mini3-VN hyperspectral imaging system.

The experiment was conducted at the experimental station of the National Agricultural Science and Technology Park, Guangzhou, located in Baiyun District of Guangdong, China (113°25’37’’E, 23°23’38’’N). This area has a subtropical monsoon climate, with an average annual temperature of 24 °C. The rice variety Meixiangzhan 2 was planted with a mean plant spacing of 25 cm. The trial comprised 30 plots, which received six different nitrogen application rates: N1 (0 kg/hm^2^), N2 (37.5 kg/hm^2^), N3 (75.0 kg/hm^2^), N4 (112.5 kg/hm^2^), N5 (150.0 kg/hm^2^), and N6 (187.5 kg/hm^2^). Each treatment was replicated five times. Phosphorus and potassium fertilizer applications were based on local standards. **Figure 2** illustrates the experimental layout.

### 2.2 Data and preprocessing

#### 2.2.1 Data collection and preprocessing of UAV hyperspectral imagery

We used the DJI Matrice M350 Pro^®^ (Shenzhen DJI Technology Co., Ltd, China) and the GaiaSky-mini3-VN^®^ push-broom imager (Jiangsu Dualix Spectral Imaging Technology Co., Ltd, China) as the UAV-borne hyperspectral imaging platform. The sensor has a spectral range of 400–1000 nm, a spectral resolution of 5 nm, and 224 bands. The UAV flew at a height of 50 m on sunny days, and a spectral calibration was performed before the flight using a standard whiteboard and gray cloth. ENVI, SpecVIEW, and HySpectralStitcher software were used for geometric correction, image stitching, and fusion. For each experimental plot, a region of interest (ROI) was manually delineated to cover the central and homogeneous rice canopy area, with careful avoidance of plot edges, visible soil, and shadows. The average spectrum was then extracted from all pixels within each ROI. Subsequently, Gaussian filtering was applied to denoise the spectral data, generating representative canopy spectral reflectance data for further analysis.

#### 2.2.2 Determination of the protein nitrogen content of rice leaves

Rice samples were collected at 5 points in an X configuration during the hyperspectral image acquisition. The leaves were washed, removed from the plant, weighed, labeled, and bagged. They were placed in an oven for drying. The initial temperature treatment was 105 °C for 30 minutes, followed by drying at 80 °C until a constant weight was achieved. The dried samples were ground and passed through a 0.25 mm sieve to determine the protein nitrogen content.

**Figure 3:**
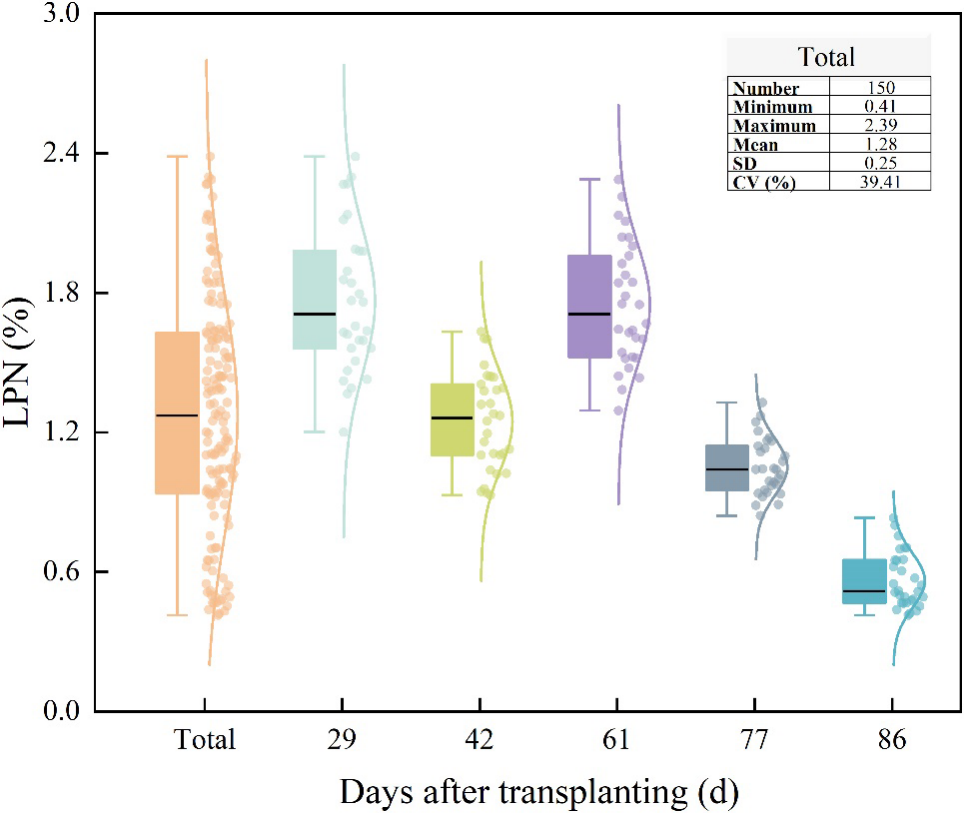
A boxplot of the rice leaf protein nitrogen.

Organic nitrogen compounds in plant tissues mainly consist of protein and non-protein nitrogen. Non-protein nitrogen comprises small molecules like amino acids and amides, along with minor inorganic nitrogenous substances, and is soluble in trichloroacetic acid (TCA). TCA was used to dissolve non-protein nitrogen and precipitate proteins. The Kjeldahl method was applied to determine the LPN (Yang et al., 2009). A total of 150 valid LPN samples were collected, forming the original dataset for model training and validation. The statistical results are presented in **Figure 3**.

#### 2.2.3 Spectral indices

Spectral indices are widely used to analyze plant nitrogen contents (Li et al., 2024). Based on relevant studies, 20 indices (**Table 1**) were chosen as preliminary variables to estimate the LPN. The indices and their equations are listed in **Table 1**.

**Table 1:** Spectral indices for estimating LPN.

Spectral index	Acronym	Equation	Reference
Normalized difference vegetation index	NDVI	(R_800_ – R_670_)/(R_800_ + R_670_)	(Chen et al., 2010)
Double-peak canopy nitrogen index	DCNI	(R_720_ – R_700_)/(R_700_ – R_670_)/(R_700_ – R_670_ + 0.03)	
MERIS terrestrial chlorophyll index	MTCI	(R_750_ – R_710_)/(R_710_ – R_680_)	(Tian et al., 2011)
Green normalized difference vegetation index	GNDVI	(R_750_ – R_550_)/(R_750_ + R_550_)	
Normalized difference red edge index	NDRE	(R_790_ – R_720_)/(R_790_ + R_720_)	
Structure intensive pigment index	SIPI	(R_800_ – R_445_)/(R_800_ – R_680_)	
Photochemical reflectance index	PRI	(R_570_ – R_530_)/(R_570_ + R_530_)	
Simple ratio pigment index	SRPI	R_430_/R_680_	
Pigment-specific simple ratio	PSSR	R_800_/R_500_	(Tao et al., 2020)
Plant senescence reflectance index	PSRI	(R_680_ – R_500_)/R_750_	
Ratio of reflectance spectra	RARS	R_760_/R_500_	
Greenness index	GI	R_554_/R_677_	
Pigment-specific normalized difference	PSND	(R_800_ – R_470_)/(R_800_ + R_470_)	
Optimized soil-adjusted vegetation index	OSAVI	1.16 ×(R_800_ – R_670_)/(R_800_ + R_670_ + 0.16)	
Red edge NDVI	RENDVI	(R_750_ – R_705_)/(R_750_ + R_705_)	(Zagajewski et al., 2018)
Simple ratio vegetation index	SR	R_750_/R_550_	(Feng et al., 2023)
Plant biochemical index	PBI	R_810_/R_560_	
Leaf chlorophyll index	LCI	(R_850_ – R_710_)/(R_850_ + R_680_)	
Normalized pigment chlorophyll ratio index	NPCI	(R_670_ – R_460_)/(R_670_ + R_460_)	
Modified simple ratio	MSR	(R_800_/R_760_ – 1)/(R_800_/R_670_ + 1)^0.5^	

*Rx* denotes the spectral reflectance at wavelength *x* nm.

### 2.3 Methods

#### 2.3.1 Determination of spectral variables for estimating LPN

An important step in estimating the LPN is selecting the characteristic variables. Following similar studies, we used XGBoost and Pearson’s correlation coefficient (PCC) to obtain the feature importance (FI) and correlation coefficients and select the spectral variables (Peng et al., 2024). Preliminary indicators were chosen after multiple trials to set thresholds. Indicators with high collinearity (∣*r*∣ > 0.8) were removed to obtain the final variables. For any pair of selected variables with an absolute correlation coefficient (∣*r*∣) ≥ 0.8 between them, the variable with a stronger correlation with LPN was retained. Where the correlations of the two variables with LPN were of comparable magnitude, the variable with higher FI was retained.

#### 2.3.2 Data augmentation

This study uses WGAN for data augmentation to ensure a sufficient sample size and prevent overfitting during regression model training. The training objective of GAN is to minimize the Jensen–Shannon (JS) divergence between the real and synthetic data distributions. If no overlap exists between the two sample distributions, a gradient of 0 may occur, leading to gradient disappearance, unstable training, and pattern collapse. WGAN resolves these issues by using the Wasserstein distance instead of the JS divergence to measure the discrepancy between the real and generated data distributions (Gao et al., 2022).

The WGAN framework consists of a generator and a discriminator. The generator seeks to produce realistic data samples from random noise inputs, whereas the discriminator aims to distinguish between samples synthesized by the generator and authentic data samples. The generator loss function of WGAN is defined as follows (Cui et al., 2024):


$$\psi_{G}=-\frac{1}{n}\sum_{i=1}^{n}D(G(z_{i}))$$

where *n* denotes the batch size, $z_{i}$ represents randomly sampled latent-space noise vectors, and $G(z_{i})$ corresponds to the output of the generator. The discriminator’s loss function is defined as follows:


$$\psi_{D}=\frac{1}{n}\sum_{i=1}^{n}[D(x_{i})-D(G(z_{i}))]$$

where $x_{i}$ denotes the samples from real data, and $D(x_{i})$ represents the discriminator’s output for authentic samples. During training, the generator enhances its generalization ability by minimizing its loss. The discriminator improves its discriminative capability by maximizing the detection accuracy of the real samples and minimizing the error rate of the generated samples. This adversarial training process results in a dynamic balance between the generator and discriminator, enabling the generator to produce realistic data samples, whereas the discriminator cannot accurately differentiate between generated and real data. The WGAN’s critical innovation is the Wasserstein distance, which is defined as (Cui et al., 2024):


$$W\left(P_{r},\;P_{g}\right)=\;{\left\|f\right\|}_{L}\leq 1\;supE_{x\sim P_{r}}\left[f\left(x\right)\right]-E_{x\sim P_{g}}\left[f\left(x\right)\right]$$

where $P_{r}$ denotes the real data distribution, $P_{g}$ represents the generated data distribution, and *f* is a Lipschitz continuous function. The discriminator of the WGAN is a Lipschitz continuous function, which is achieved by applying weight regularization to the parameters. In summary, the WGAN improves the training performance by optimizing the Wasserstein distance, enabling the generator and discriminator to learn the features of the data distribution more stably and generate higher quality samples. The model was trained for a maximum of 200 epochs with a batch size of 16, using the Adam optimizer with $\beta_{1}$ = 0 and $\beta_{2}$ = 0.9. The learning rate was set to 0.0001 for the generator and 0.0002 for the discriminator, with the discriminator updated five times per generator update.

#### 2.3.3 Model construction and validation

The screened variables were used as independent variables, and the LPN was used as the dependent variable. Four algorithms were used to create the relationship models between the variables and the LPN: MLR, PLSR, SVM, and KNN. More details are provided in the literature (Liu et al., 2022; Lin et al., 2024; Yang et al., 2025). The original 150 samples were partitioned into a training set (120 samples, 80%) and a test set (30 samples, 20%) at a 4:1 ratio. This partitioning was performed prior to feature selection to avoid test set bias. All models were developed using the selected variables and this predefined data split, and model performance was evaluated on the held-out test dataset. Accuracy assessment (using the coefficient of determination (R^2^) and the root mean square error (RMSE)) was performed using the test dataset to determine the most accurate algorithm for estimating the LPN. All model implementations were carried out in Python software.

#### 2.3.4 Model explanation

The SHAP is a game theory method for interpreting machine learning models (Lundberg and Lee, 2017). It assesses the variables’ impacts by calculating the marginal contributions of the variables to the model output, evaluating the entire dataset and individual samples. SHAP generates the Shapley value for the features. It performs weighting, and a linear function (*g*) with binary variables is obtained to estimate the objective function (*f*), which is expressed as (Li et al., 2025; Zhong et al., 2024):


$$g(x)=f(x)=\emptyset_{0}+\sum_{i=1}^{M_{0}}\emptyset_{i}$$

where $M_{0}$ is the number of features in the objective function of the black-box model ( f(x)); $\emptyset_{0}$ is the average of the predicted values of the objective function for all samples; and $\emptyset_{i}$ is the Shapley value of the feature, which is denoted as:


$$\emptyset_{i}=\sum_{S\subseteq \{M/x_{i}\}}\frac{|S|!(|M|-|S|-1)!}{|M|!}\{f(x_{S\cup \{i\}})-f(x_{s})\}$$

where *M* denotes the full set of features; *S* indicates the subset of features; $f(x_{S\cup \left\{i\right\}})$ and $f(x_{s})$ denote the model outputs when feature $x_{i}$ is modeled and not modeled, respectively, for various feature combinations; $\frac{|S|!(|M|-|S|-1)!}{|M|!}$ represents the probability corresponding to the feature combinations. More details are provided in the literature (Lundberg et al., 2020).

## 3 Results

### 3.1 Variable selection

**Figure 4:**
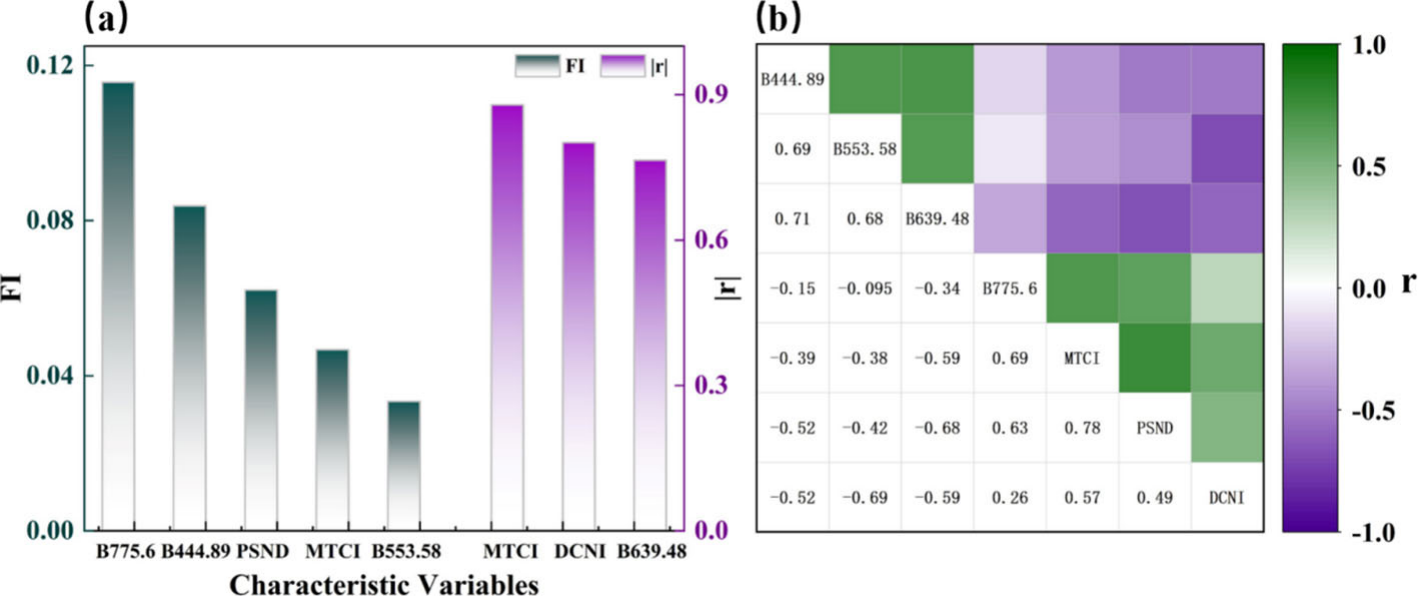
**(a)** FI and **(b)** r of the selected indicators used to estimate LPN.

PCC and XGBoost were employed to select the feature variables. Systematic experiments were conducted. The thresholds for feature importance (FI > 0.02) and Pearson correlation coefficient (∣*r*∣ > 0.75) were determined through iterative experimentation to balance variable relevance with model complexity. Correlation analysis was utilized to mitigate multicollinearity among the selected variables, with a threshold of ∣*r*∣ < 0.8 for exclusion. As shown in **Figure 4**, the two algorithms identified 5 (B_775.6_, B_444.89_, B_553.58_, pigment-specific normalized difference (PSND), and MERIS terrestrial chlorophyll index (MTCI)) and 3 (B_639.48_, double-peak canopy nitrogen index (DCNI), and MTCI) variables, respectively, with MTCI being a common variable selected by both methods.

### 3.2 Augmentation dataset

**Figure 5:**
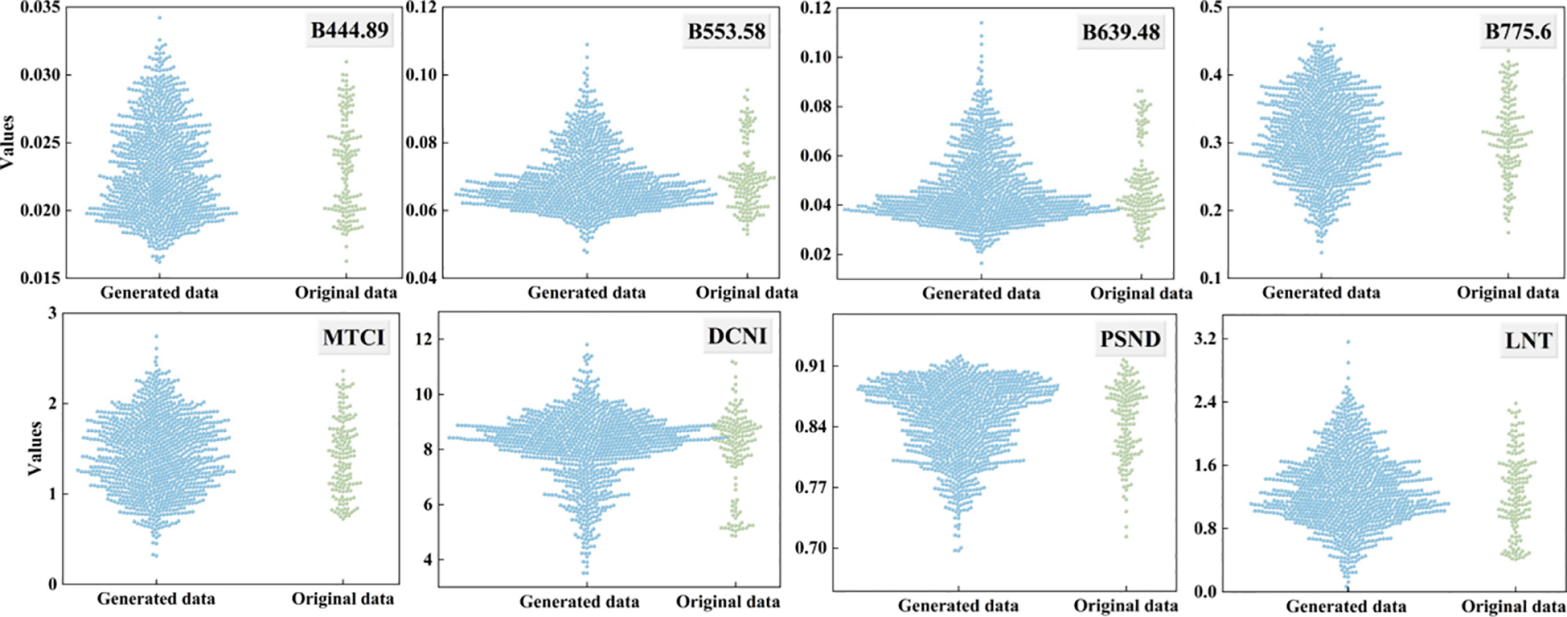
The values of the original and generated spectral variables.

WGAN was employed to augment the sample dataset to obtain sufficient samples for training the machine learning models. To prevent data leakage, the WGAN was trained exclusively on the training set, generating 1000 synthetic samples to augment the 120 original training samples, while the independent test set was strictly reserved for final validation. Spectral variables were employed to evaluate the efficacy of the WGAN model in increasing the sample size (see **Figure 5**). The distribution of the generated sample dataset closely resembled that of the original dataset, indicating the preservation of data features. However, the generated dataset did not have the exact distribution of the original dataset because the WGAN model synthesized new samples by learning the underlying distribution rather than duplicating existing data. Consequently, the WGAN-augmented dataset was suitable for the subsequent development of the LPN estimation models.

### 3.3 Model construction and accuracy evaluation

**Figure 6:**
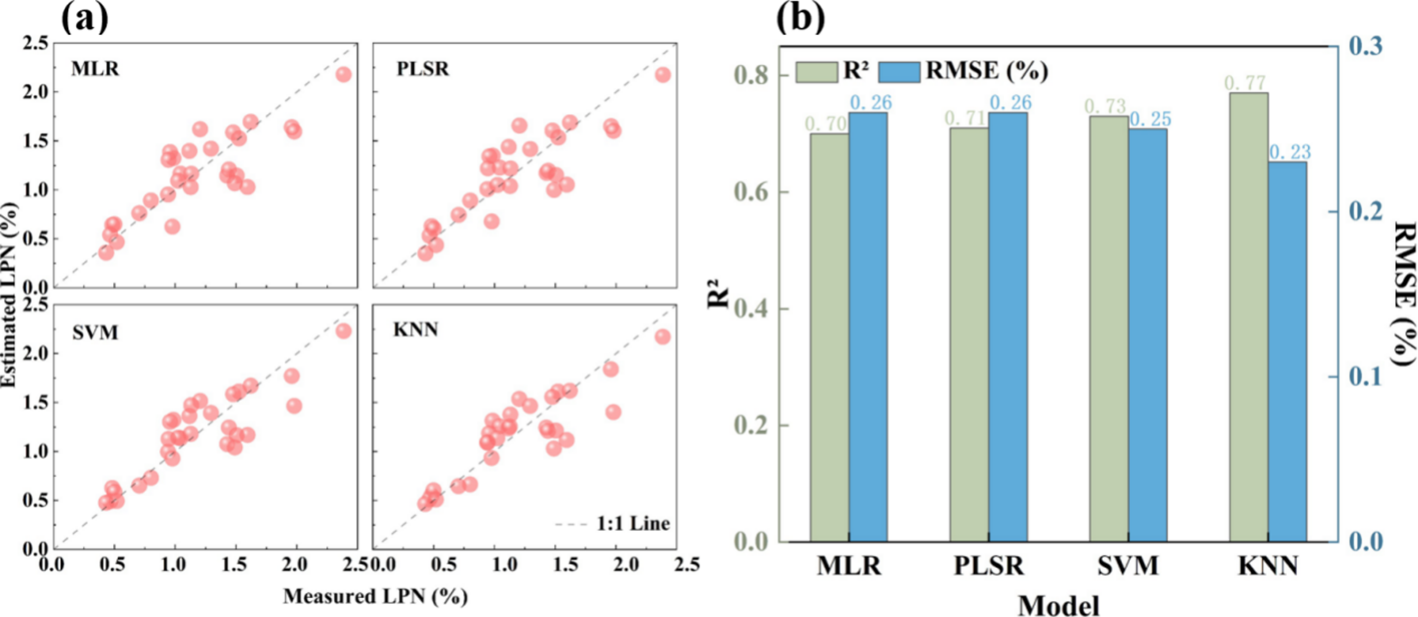
**(a)** Scatterplots of measured and estimated LPN and **(b)** estimation accuracies based on the test dataset.

The scatterplots of the measured and estimated LPN derived from the PLSR, MLR, SVM, and KNN algorithms are shown in **Figure 6**. The results indicate that nonlinear machine learning methods (SVM and KNN) outperformed linear regression models (PLSR and MLR) in estimation accuracy. The KNN-based estimation model demonstrated the best predictive performance, with a test R² of 0.77 and an RMSE of 0.23%, highlighting the significant advantages of machine learning models in capturing complex spectral-physiological relationships.

**Figure 7:**
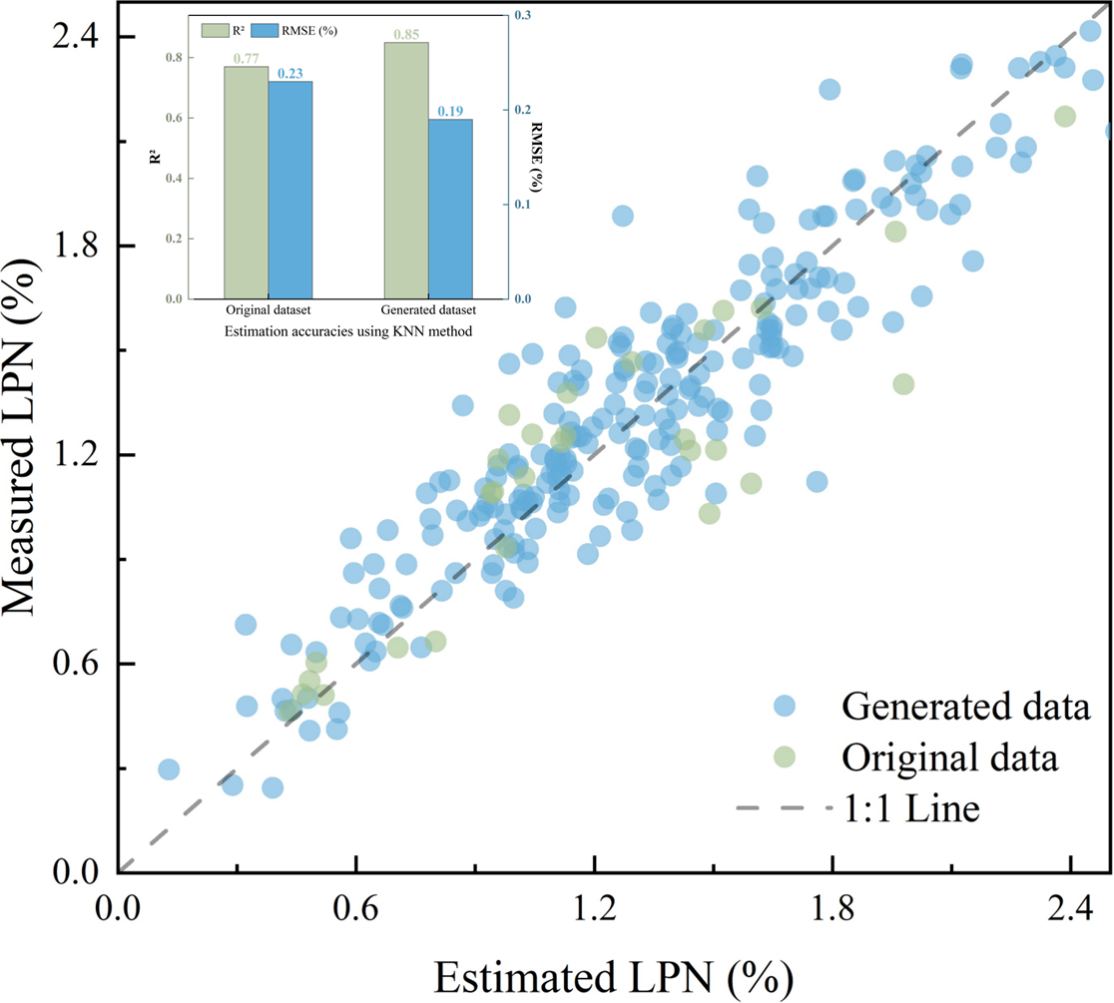
Scatterplots of measured and estimated values and estimation accuracies of the KNN method using the generated data and the original dataset.

The optimal model (KNN) was used to conduct an experiment to assess the effect of adding generated data on model estimation, as shown in **Figure 7**. The model that used the generated data exhibited points closer to the 1:1 line and a 10.39% improvement in the R² value than the model with the original data. This finding suggests that data augmentation improved the performance of machine learning models, particularly in cases with small sample sizes.

### 3.4 Contribution of variables to model output

**Figure 8:**
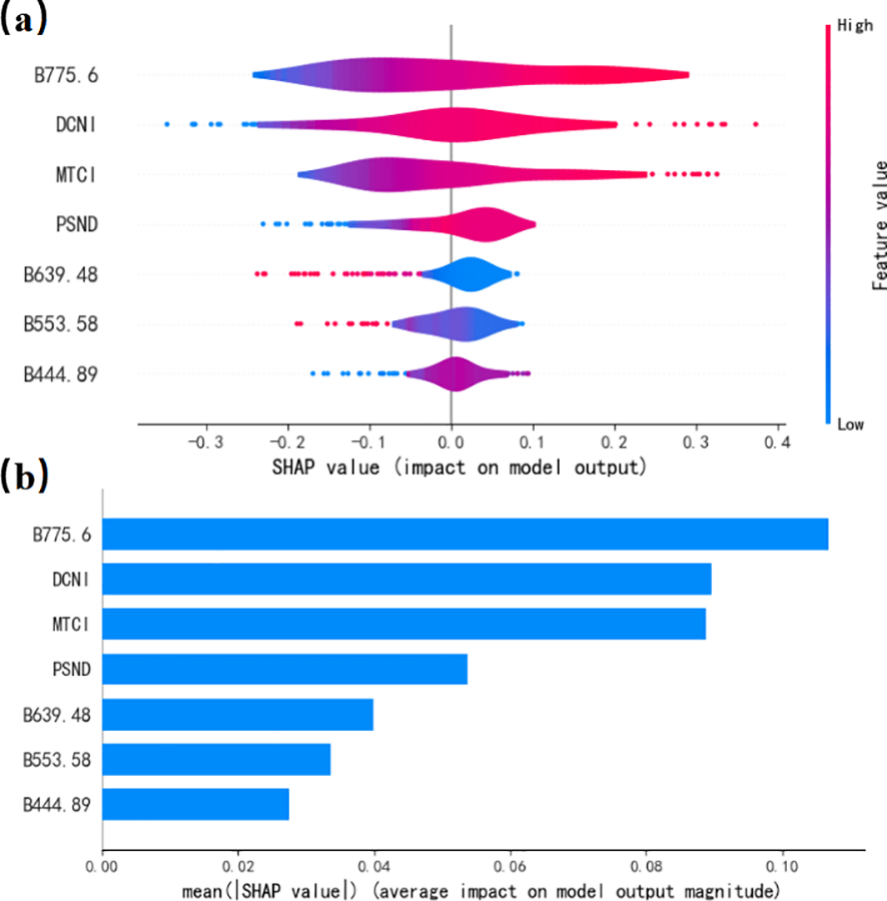
Feature importance **(a)** and summary plot **(b)** of the KNN model (SHAP values).

The contribution of the variables to the model output was ranked using summary plots derived from the SHAP tool (**Figure 8**). The SHAP values quantify the impacts of the features on model estimations. Positive SHAP values indicate a positive contribution to the model output, whereas negative values indicate a negative contribution. The points in **Figure 8** represent the values of the features for an individual sample. The color gradient, ranging from red to blue, reflects the magnitude of the feature value, and the position on the axis corresponds to the SHAP value for that feature in that sample. The results show that B775.6 contributed the most to the model output. **Figure 8b** shows that B775.6, DCNI, and MTCI have the three highest importance values in the LPN estimation model. Their average absolute SHAP values exceed 0.08, indicating they have the largest influence on the model output.

**Figure 9:**
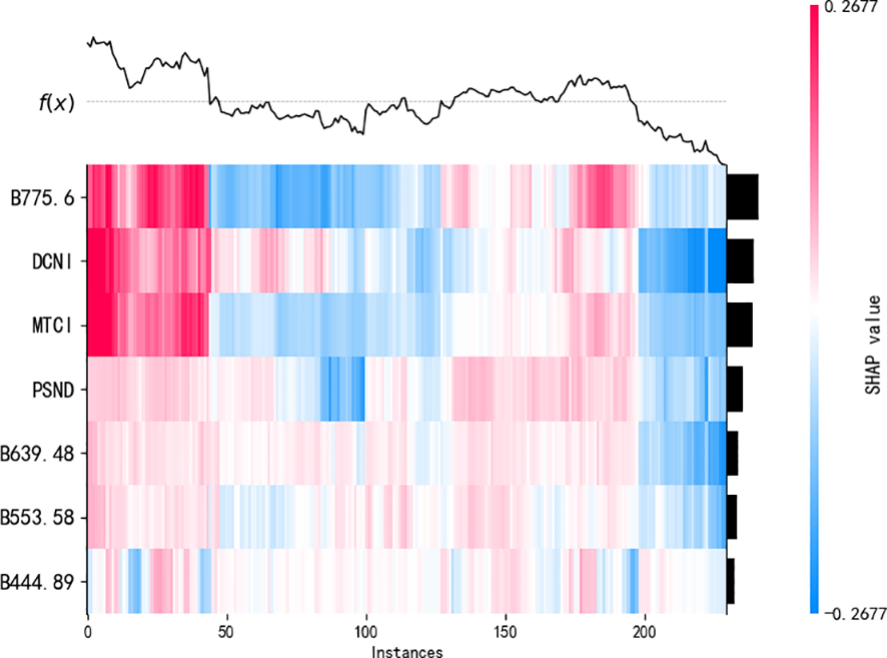
Heatmap of the SHAP values.

A heatmap of the SHAP values was created to clarify the association between the interpretable outcomes and the LPN under optimal conditions. As shown in **Figure 9**, the distribution and magnitude of the feature contributions varied across the samples, with different contents under the best scenarios. The sample numbers in the x-axis direction are arranged according to hierarchical clustering based on their explanatory similarity, and the features are displayed on the y-axis. The SHAP values are represented on a color scale. The values on the x-axis reflect the cumulative SHAP values for each sample. They are the output of the explanatory analysis. Hierarchical clustering and marginal contribution analyses of the importance value demonstrated that the LPN estimation model showed a multifactorial synergistic effect. B775.6 and DCNI had the largest influence on the model output.

## 4 Discussion

Due to the increase in global population, agricultural production faces the significant challenge of reducing the environmental impact of excessive nitrogen fertilizer use while maintaining high crop yields (Shen et al., 2024). The global nitrogen input to farmland is approximately 200 million tons, but its utilization efficiency is less than 50 percent. Thus, more than half of the applied nitrogen is not absorbed by crops but is lost to the environment, adversely affecting air quality and water resources (Zhang et al., 2021; Gu et al., 2023). Nitrogen loss not only threatens environmental safety but also poses potential risks to public health. Therefore, obtaining accurate information on rice nitrogen status is crucial for optimizing nitrogen fertilizer applications and improving nitrogen utilization efficiency (Wang et al., 2022). UAV-based hyperspectral imagery is critical for monitoring the nitrogen status of rice (Li et al., 2024).

We utilized UAV hyperspectral imagery and multiple models to estimate the LPN. The model performance analysis reveals two key findings. First, KNN outperformed other models owing to its non-parametric local learning approach, which effectively captures the complex nonlinear relationships in hyperspectral dataset without overfitting given the limited sample size. Second, the WGAN-based data augmentation improved performance by learning the underlying data distribution and generating high-quality synthetic samples. These samples fill sparse regions in the feature space, providing KNN with more comprehensive local instances that enhance local feature neighborhoods. The KNN model combined with WGAN-generated data outperformed the model trained on the original data, suggesting that the accuracy of machine learning models substantially depends on sample size, whereas WGAN effectively mitigates data scarcity by generating sufficient training data.

While the results demonstrate the effectiveness of WGAN-based data augmentation in improving LPN estimation accuracy, we acknowledge that alternative strategies, such as the use of strong ensemble learners like Random Forest or AdaBoost, may also yield satisfactory performance with the original dataset. A systematic comparison between these ensemble methods trained on the original dataset and the proposed WGAN-augmented model would provide valuable insights into whether data augmentation offers a distinct advantage over algorithm selection. We therefore plan to investigate this comparison in future work, extending our analysis to include a broader range of models and further validating the conditions under which data augmentation yields the greatest improvement.

We employed the SHAP tool to interpret the estimation results of the machine learning model, revealing the variables’ contributions to the model output. This method identified the influences of key features and provided an intuitive explanation of the model’s decision-making. However, this study primarily focused on model interpretability and did not use the SHAP values to optimize the estimation model. In future research, we will build on the SHAP analysis results to improve model performance through feature selection, model refinement, and multi-source data integration.

Despite the progress achieved in this study, certain limitations remain. First, the model was developed and validated with data from a single year and rice variety, which may limit its generalizability. Future work will involve validating and refining the method across multiple years, diverse geographical locations, and additional rice varieties. Techniques such as transfer learning will be explored to enhance model generalizability under these varying conditions. Furthermore, the limited sample size (n=150), constrained by weather and cost, precluded an evaluation of model performance across different growth stages and nitrogen application rates. Future research will therefore focus on collecting more comprehensive datasets across multiple growth stages and nitrogen treatments to enable a more robust assessment of model generalizability and stability under diverse field conditions.

Second, while this study achieved accurate point-scale LPN estimation, it did not address the spatial distribution of nitrogen or its interactions with soil, climate, and other environmental factors. Future research will apply the developed model to UAV hyperspectral imagery to generate spatial distribution maps of LPN across the study area. These maps will then be integrated with soil fertility information and crop fertilizer demand to develop precision fertilization prescription maps. Such efforts will provide scientific and technical support for precision fertilization in rice cultivation and promote sustainable agricultural development.

## 5 Conclusion

Accurate monitoring of rice nitrogen is crucial for optimizing nitrogen fertilizer applications and improving rice yield. We utilized data augmentation algorithms and machine learning models to develop a model for estimating the LPN. We employed SHAP to analyze the variables’ contributions to the model output. The results demonstrated that the KNN model outperformed statistical regression methods (MLR and PLSR), with an R² of 0.77. The KNN model with the WGAN-generated data outperformed the model trained on the original data, resulting in a 10.39% improvement in the R² value. This result indicates that data augmentation has the potential to improve the performance of machine learning models when small sample sizes are used. The SHAP analysis revealed that the final model exhibited multifactor synergistic effects. The B_775.6_, DCNI, and MTCI had the largest influences on the model output. These findings provide valuable insights into field management and fertilizer applications in rice cultivation.

## Data Availability

The original contributions presented in the study are included in the article/supplementary material. Further inquiries can be directed to the corresponding author/s.
